# Expression of Human CD4 and chemokine receptors in cotton rat cells confers permissiveness for productive HIV infection

**DOI:** 10.1186/1743-422X-6-57

**Published:** 2009-05-14

**Authors:** Jorge CG Blanco, Lioubov M Pletneva, Lindsay Wieczorek, Dimple Khetawat, Tzanko S Stantchev, Christopher C Broder, Victoria R Polonis, Gregory A Prince

**Affiliations:** 1 Virion Systems Inc., 9610 Medical Center Drive, Suite 100, Rockville, Maryland 20850, USA; 2 The United State Military HIV Research Program, Rockville, Maryland 20850, USA; 3 Department of Microbiology and Immunology, Uniformed Services University of the Health Sciences, Bethesda, Maryland 20814, USA

## Abstract

**Background:**

Current small animal models for studying HIV-1 infection are very limited, and this continues to be a major obstacle for studying HIV-1 infection and pathogenesis, as well as for the urgent development and evaluation of effective anti-HIV-1 therapies and vaccines. Previously, it was shown that HIV-1 can infect cotton rats as indicated by development of antibodies against all major proteins of the virus, the detection of viral cDNA in spleen and brain of challenged animals, the transmission of infectious virus, albeit with low efficiency, from animal to animal by blood, and an additional increase in the mortality in the infected groups.

**Results:**

Using *in vitro *experiments, we now show that cotton rat cell lines engineered to express human receptor complexes for HIV-1 (hCD4 along with hCXCR4 or hCCR5) support virus entry, viral cDNA integration, and the production of infectious virus.

**Conclusion:**

These results further suggest that the development of transgenic cotton rats expressing human HIV-1 receptors may prove to be useful small animal model for HIV infection.

## Background

All vaccines and therapeutic strategies against HIV-1 must be evaluated in animal models in order to select those that may be appropriate to further advance into clinical trials in humans. It is the goal of such animal models to recreate critical aspects of viral replication, transmission and pathogenesis as seen in humans. The most utilized animal models for developing anti-HIV-1 vaccines and drugs have been the non-human primate (NHP) systems[1]. NHPs do not efficiently replicate HIV-1 due to host restriction factors[2,3]. Thus, current NHP models are based on infection of different species of macaques, or less often chimpanzees, with lentiviruses of non-human primates, i.e. simian immunodeficiency viruses (SIVs), or with chimeric viruses, i.e. simian-human immunodeficiency viruses (SHIVs). Although substantial knowledge has been gained from modeling HIV-1 infection in NHP, the high expenses, the ethical concerns associated with performing experiments in primates, and their outbred nature continue to represent important obstacles to accelerate the development of new vaccines and therapies.

Since small laboratory animals are unable to replicate HIV-1 due to a series of species-specific blockages including entrance and viral gene transcription[4], intensive efforts were directed to modify these models to render them permissive for HIV-1 infection. Hence, humanized mouse models, namely severe combined immunodeficiency (SCID) mice in which human peripheral blood mononuclear cells are injected peritoneally (hu-PBL-SCID), or in which surgical engraftment of human fetal hematopoietic tissue, namely thymus and liver, is implanted under the kidney capsule (hu-Thy/Li-SCID), have been used to achieve productive HIV-1 infection[5,6]. However, these are technically very challenging studies, are time consuming, and do not fully recapitulate HIV-1 infection within the context of an intact immune system.

Binding of HIV-1 envelope (*Env*) to both CD4 and an appropriate member of the seven-transmembrane G-protein-coupled receptor superfamily are necessary for the efficient entry of HIV-1[7,8]. Several different chemokine receptors (CCR2b, CCR3, CCR5, or CXCR4) or orphan chemokine receptor-like molecules (STRL33, GPR1, GPR15, V28, APJ) may participate in HIV-1 entry, but hCXCR4 and hCCR5 are the principal co-receptors for X4 (T-cell line-tropic) or R5 (macrophage-tropic) isolates, respectively. Blocking and down-regulation of these two chemokine receptors are ways by which their physiological ligands or modified analogues can prevent or reduce HIV-1 entry[9].

The characterization of HIV-1 receptors prompted the development of several transgenic animals expressing the human receptors for HIV-1, including mice[10,11], rats[12], and rabbits[13,14]. The outbred transgenic rat model, expressing hCD4 and CCR5 on lymphocytes, macrophages, and microglia, have been recently shown to be promising for testing antiviral compounds targeting HIV-1 entry and reverse transcription, despite the transient levels of HIV-1 replication[15]. These results are encouraging for the anti-HIV-1 drug development field and further validate the transgenic approach to develop small animal models for HIV-1 research.

Previously, we and others [16-19] have shown evidence of HIV-1 infection in two cotton rat species (*Sigmodon hispidus and S. fulviventer*). In one study [16] cotton rats inoculated with HIV-1 developed detectable amounts of proviral DNA in peripheral blood mononuclear cells (PBMC). Virus inoculation induced a distinct and characteristic HIV-1 antibody response that in some animals included the elicitation of antibodies that recognized all the major HIV-1 antigens, and that persisted for at least 52 weeks post-infection.

In another series of studies, Rytik and collaborators [17-19] infected cotton rats (*S. hispidus*) with a Russian isolate of HIV-1. Analysis of the infected animals showed that 75% of the samples from spleen and half of the samples from brain obtained 3 months post-infection contained proviral DNA, whereas all the samples from both tissues obtained 6 months post-infection were positive for proviral DNA. Taken together, these results suggest that low levels of productive infection may occur in cotton rats.

We hypothesized that the lack of specific HIV-1 receptors on the surface of cotton rat cells strongly reduces viral entry, and although additional intracellular obstructions may exist, entry appears to be the major feature responsible for the restricted viral replication seen *in vivo*. In this new set of experiments we demonstrate that primary cotton rat macrophages, transfected with a HIV-1 backbone plasmid encoding a luciferase reporter gene, are able to support HIV-1 gene expression. Furthermore, by producing a series of cotton rat cell lines expressing human CD4 and CXCR4 or CCR5, we were able to demonstrate that CD4 and co-receptor expression was sufficient to enhance HIV-1 entry, DNA integration, and production of infectious viral particles in cotton rat cells.

## Results

### Isolation of cotton rat cells expressing human CD4 and HIV-1 co-receptors

**Figure 1:**
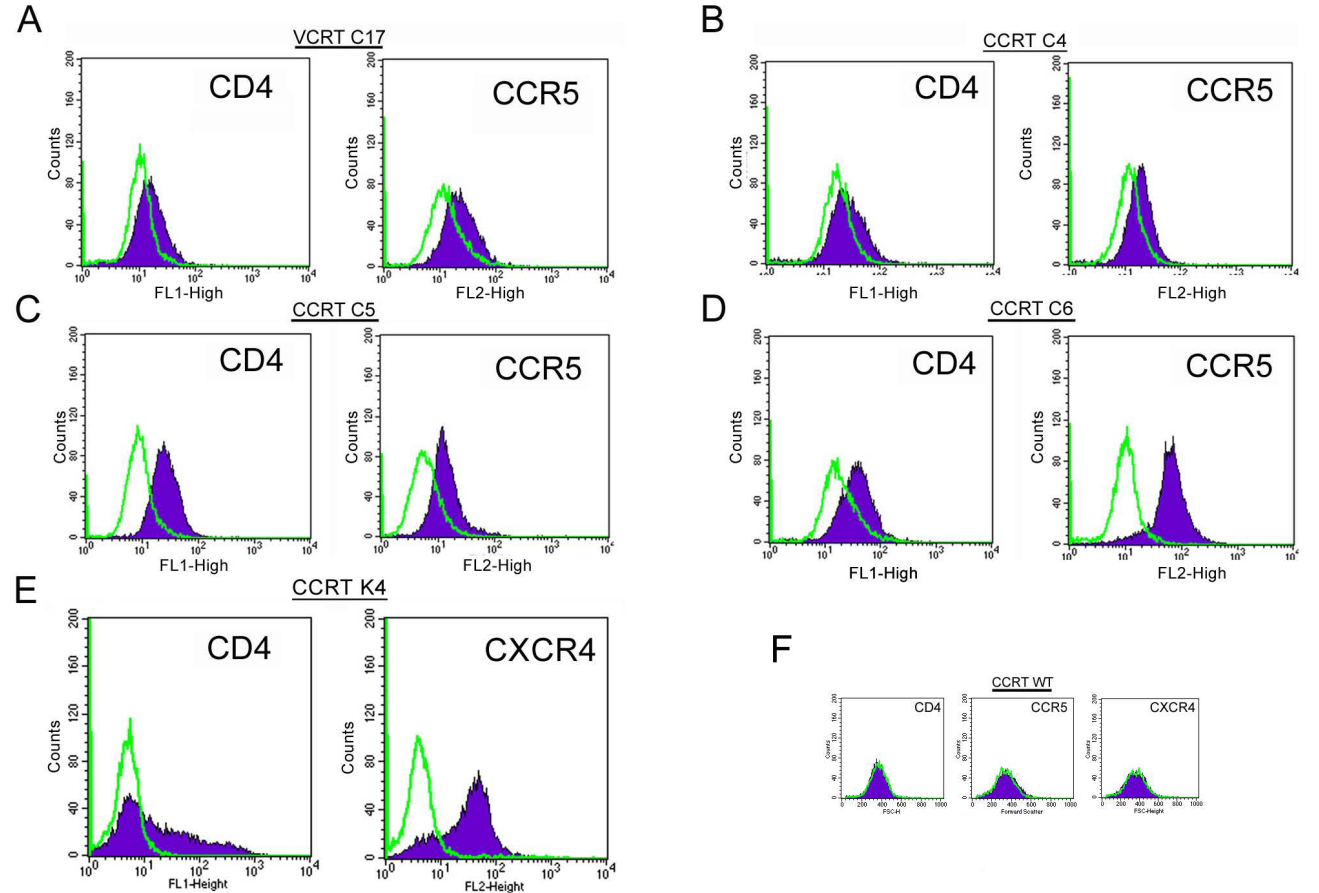
**Isolation and cloning of cotton rat cells expressing human co-receptors for HIV-1**. (A), VCRT C17; (B), CCRT C4; (C), CCRT C5; (D), CCRT C6; and (E), CCRT K4, were established which expressed human CD4 molecule, with human CXCR4 or CCR5, as indicated. The expression of these surface molecules was measured by FACs using fluorescent antibodies against the indicated molecules (CD4, clone RPA-T4; CXCR4, clone 12G5; CCR5, clone 2D7) (filled profile) and compared to the staining obtained of the same cells with control antibody isotypes (empty profiles). (F) Staining profile of parental CCRT cells with the above mentioned antibodies, as indicated.

To demonstrate that cotton rat cells lack the strong transcriptional blockages for HIV-1 replication that other rodents have and that virus entry is the major hurdle for HIV-1 to replicate in this species, clones of the cotton rat cell lines VCRT (C17) and CCRT (C4, C5, and C6) expressing hCD4 and hCCR5 molecules were established using a pleiotropic retrovirus expression system and analyzed by flow cytometry. Measurable levels of both receptors were expressed in the entire population of these cells as indicated by the shift in their fluorescent intensity compared to cells stained with a control antibody isotype (Fig. 1A–D). In addition a clone of CCRT cells expressing both hCD4 and hCXCR4 (K4) was also established using pCDNA3.1 expression vectors (Fig. 1E). The proportion of double expressor cells in the population of this clone was ~32% during early passages and this proportion progressively decreased with successive passages. Thus, an early passage of this clone (passage #3) was used for the subsequent infection experiments. No increase in fluorescence was detected when parental CCRT or VCRT cells were stained with any of the anti co-receptors' antibodies (Fig. 1F and data not shown).

### Cotton rat cells expressing human CD4 and HIV-1 co-receptors are permissive for infection with HIV-1

To assess whether the expression of HIV-1 receptor complex on cotton rat cells render them permissive for viral infection, the different receptor expressing clones and the parental cell lines were challenged with different HIV-1 isolates and the production of p24gag was measured in supernatants after infection (Table 1). CCRT cells expressing hCD4 and hCXCR4 (K4 clone) were positive for the production of p24gag after challenge with the MN (X4-tropic) isolate, but produced only basal levels of p24gag when challenged with the BAL (R5-tropic) isolate. On the contrary, CCRT cells expressing hCD4 and hCCR5 (C6 clone) were positive for the production of p24gag when infected with the R5-tropic isolates (BAL and US1), but not when infected with the X4-tropic isolate MN. As expected, parental CCRT cells only showed marginal levels of production of p24gag after incubation with the different isolates.

**Table 1:** p24gag Concentration (pg/ml) in Supernatants^a ^of Cotton Rat Cells infected with Different HIV-1 Isolates.

Cell Clones^b^		Virus Isolates^c^		
	Mock	BAL	MN	US1

CCRT	<15	331 ± 20	110 ± 14	54 ± 2

CCRT K4	<15	33 ± 6	25095 ± 3191	n.d.^d^

CCRT C6	<15	18477 ± 1119	35 ± 2	16635 ± 1743

VCRT	<15	142 ± 12	89 ± 9.5	46 ± 2

VCRT C17	<15	1072 ± 37	106 ± 8	40 ± 3

^a^Supernatants of each infection experiments were tested for the presence of p24gag in duplicates at several time post infection (day 1 to 24) and the peak concentration is presented. Results represent means ± SD of at lease two experiments.

^b^Cotton rat cell clones expressing human CD4 with CXCR4 (CCRT K4), or with CCR5 (CCRT C6 and VCRT C17).

^c^HIV-1 viral isolates X4-tropic (MN) and R5-tropic (BAL and US1).

^d ^not determined.

To further examine whether the permissiveness of the cotton rat for HIV-1 replication was not due to a unique characteristic of the CCRT cell line, we engineered the cotton rat VCRT cell line to express hCD4 and hCCR5, and challenged them with different HIV-1 isolates. As shown in Table 1, the VCRT clone C17 showed significantly higher levels of expression of p24gag (~1 ng) than those found in the control parental VCRT line when infected with the BAL isolate. These results demonstrate that cotton rat cells expressing HIV-1 co-receptor complexes are permissive for HIV-1 replication. Furthermore, since these clones were originally derived from different cotton rat cell lines, these data suggest that HIV-1 permissiveness of cotton rat cells is not a cell type specific attribute.

### Kinetics of p24gag production by cotton rat transfectant cells after HIV-1 infection

**Figure 2:**
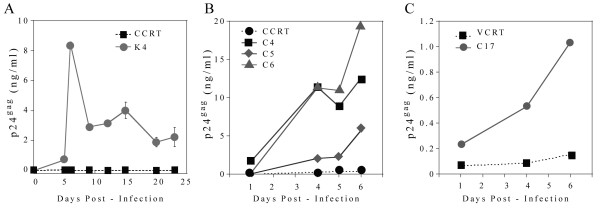
**Kinetic of the expression of p24gag in supernatants of cotton rat cell clones after HIV-1 infection**. (A), CCRT parental cell line and the derived clone K4 which expresses hCD4 and hCXCR4 infected with MN isolate; (B), CCRT parental cell line and the C4, C5, and C6 derived clones expressing hCD4 and hCCR5 infected with HIV-1 BAL isolate; (C), VCRT parental cell line and the derived C17 clone expressing human CD4 and CCR5 infected with HIV-1 BAL isolate.

Next, we infected the engineered cotton rat CD4 clones to define the time course of p24gag production (Fig. 2). When the CCRT K4 clone was infected with HIV-1 MN, a peak in p24gag concentration was detected six days post-infection, after which the amount of p24gag decreased and subsequently was maintained at 2–4 ng/ml levels until the last sample was taken on day 23 (Fig. 2A).

Infection of the CCRT clones (C4, C5 and C6) or the VCRT clone C17, which express hCD4 and hCCR5, with the HIV-1 BAL isolate showed similar results (Fig. 2B and 2C). Some of the clones showed detectable levels of p24gag in their supernatant as early as one day post infection (CCRT C4, Fig. 2B; and VCRT C17, Fig. 2C), whereas the CCRT C5 and C6 showed detectable p24gag expression starting on day 4. Later than day 6 post-infection, the levels of p24gag were reduced to about 50% of the peak level, which paralleled the increase in cell mortality (data not shown). Although the concentration of p24gag in the supernatant of VCRT C17 infected cells was lower than those obtained with the CCRT-derived clones, its concentration was at all times higher than those obtained from supernatants of the infected parental VCRT line. These data further demonstrate that cotton rat cells expressing the human receptor complex for HIV-1 become permissive for HIV-1 infection and produce peak amounts of p24gag in their supernatant 6 days post-challenge.

### Detection of infectious virions in supernatants of HIV-1 infected cotton rat cells

**Figure 3:**
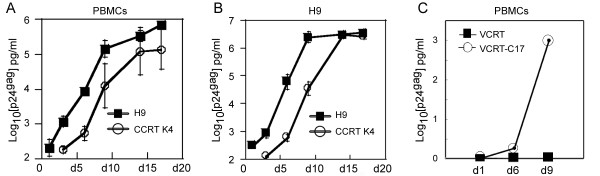
**HIV-1 virus stocks produced in cotton rat cells infect human cells**. Kinetic of p24gag expression by human PBMC (A), or by the human H9 T cell line (B), when infected with a stock of HIV-1 MN grown from CCRT K4 cells. The growth kinetic of the virus in these stocks is compared with the original MN stock grown on H9 cells. (C) Kinetics of P24gag expression by human PBMC infected with HIV-1 BAL isolate generated in the cotton rat cell line VCRT C17.

**Figure 4:**
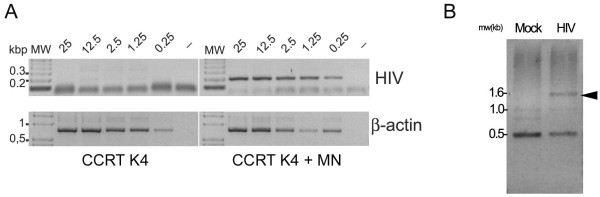
**Detection of episomal cDNA and isolation of cotton rat-HIV-1 chimeric DNA sequence from HIV-1 infected cotton rat cells**. (A), CCRT K4 cotton rat clone infected with HIV-1 MN and incubated for 4 days before isolation of genomic DNA for the detection of episomal cDNA. Numbers on the top represent the amounts of DNA (ng) used as input for each PCR reaction. HIV and β-actin panels indicate the fragments amplified in each panel. (B), DNA preparations from infected (HIV) and uninfected (mock) CCRT K4 cells were subjected to PCR amplification using one primer targeting a genomic cotton rat sequence and another primer targeting the sequence of HIV-1 MN LTR. The indicated band (arrow) was isolated, cloned and its sequence (GenBank AY703985) confirmed the integration of HIV-1 into the cotton rat genome.

To demonstrate that the p24gag protein released into the supernatants of infected cotton rat cells represented infectious HIV-1 virus, supernatants obtained on day 6 post challenge were used to infect HIV-1 permissive human cells. p24gag positive supernatants from CCRT K4 cells infected with MN virus (containing ~8 ng/ml of p24gag) or from VCRT C17 cells infected with BAL virus (containing ~1 ng/ml of p24gag) were collected and used for infection of human PBMC and H9 cells (CCRT K4 derived virus, Fig. 3A and 3B respectively) or only PBMC (VCRT C17 derived virus, Fig. 3C). In some experiments, equivalent amounts of p24gag protein (8 ng/ml) from stocks of the strain MN prepared in H9 cells were tested as positive control (Fig. 3A and 3B). Detectable amounts of p24gag were obtained as early as day 6 post transfer of all the cotton rat-derived stocks of virus (Fig. 3) but not when transferred stocks were prepared from mock inocula (data not shown and Fig. 3C). A sharp increase in the production of p24gag by the recipient human cells occurred on day 9 and continued increasing to reach a plateau when the stocks used were derived from CCRT K4 cells infected with MN (Fig. 4A and 4B).

A delay in the kinetics of expression of p24gag was observed in stocks of HIV-1 derived from cotton rat cells compared to H9-derived HIV-1 stocks (Fig. 3A and 3B). This delay most likely represents lower p24gag:TCID_50 _ratios in the stocks prepared in the cotton rat and might indicate that the virus passed through cotton rat cells became less permissive to human cells. Overall, the comparable infectivities obtained with human and cotton rat cell-derived virions demonstrate that fully mature, infectious HIV-1 particles can be efficiently synthesized, assembled and released from cotton rat cells.

### Detection of episomal and integrated HIV-1 cDNA in infected cotton rat cells

In order to detect the presence of HIV-1 episomal cDNA in infected cotton rat cells, total genomic DNA was isolated from HIV-1 MN-infected or uninfected CCRT K4 cells and subjected to PCR amplification using primers derived from the sequence of the LTRs of the HIV-1 MN isolate. A unique fragment of the expected length (197 bp) and identity was found only in those DNA samples obtained from infected cotton rat cells (Fig. 4A).

Finally, we examined whether the HIV-1 cDNA was able to integrate into the genome of the infected cotton rat cells. For this purpose, we analyzed total genomic DNA from infected and uninfected cotton rat cells by PCR using one primer that anneals to a known repetitive sequence in the cotton rat genome (LINE-1 like sequence) and another primer that anneals to the Long Terminal Repeats (LTRs) sequence of the HIV-1 MN isolate. After amplification, the resulting DNA products were separated by agarose gel electrophoresis, and a fragment of ~1.6 Kbp detected only in infected cells was isolated and cloned (Fig. 4B, arrow). Sequencing revealed that this fragment had a chimeric structure which included 650 bp of the 5' LTR of HIV-MN and 950 bp of the cotton rat repetitive LINE-1 sequence (GenBank accession no. AY703985), evidencing insertions of viral cDNA into the genome of cotton rat cells. These data clearly demonstrate that cotton rat cells are able to support the integration of viral cDNA into their genome.

## Discussion

More than twenty five years following the first description of AIDS and the subsequent isolation of HIV-1, there remains a paucity of suitable small animal model systems to study HIV-1 infection and pathogenesis. The absence of the specific HIV-1 receptors on the cell membrane of these small animal models has been a major obstacle for the study of productive viral entry and infection. To circumvent this hurdle, mice expressing hCD4 and hCCR5 or hCXCR4 were developed[10,11]. Preliminary results with these mice were at first exciting because the expression of the transgenes promoted viral entry, but these mice did not support robust viral replication[11]. These results were partly explained by differences between the human and the mouse cyclin T1 (CycT1), which is an important cellular component of the pTEFb transcription factor complex responsible for transcription from the viral HIV-LTR[20,21]. Thus, the humanized mouse models seem to be the best alternative for HIV-1 pathogenesis studies using this rodent[5,6]. In addition, transgenic rats (*Rattus norvegicus*) expressing hCD4 and hCCR5 were developed[12]. Despite only transient HIV-1 replication *in vivo*, this model was recently proposed as a system to allow rapid clinical testing of antiviral compounds that target virus entry or reverse transcription[15].

In our previous study[16], provirus was consistently detected in PBMC preparations and other tissues (spleen, thymus and bone marrow) from infected cotton rats. The virus induced a strong, specific and long-lasting immune response that was maintained up to 1 year post-infection. Although not demonstrated by direct culture of PBMC or tissues from infected animals, infectious virus replicated at a low level in cotton rats since viral cDNA was detected by PCR in animals that underwent three serial passages of blood. In addition, Rytik and collaborators[19] detected viral cDNA in spleen and brain of all the infected animals 6 months post-infection, showed increase in mortality (17%), and found morphological changes in cells of the CNS.

We have hypothesized that the expression of HIV-1 receptors on the surface of cotton rat cells would allow them to efficiently replicate HIV-1 virus. Different clones of cotton rat cell lines expressing measurable levels of hCD4 and hCCR5 or hCXCR4 were then produced and cultured with infectious HIV-1. The data presented here clearly indicate the productive infection of these cotton rat cells when infected with well characterized HIV-1 strains (MN, BAL and US1). Indicators of productive infection were [1] p24gag detection in the supernatant of infected cells, [2] detection of episomal viral cDNA in infected cells, [3] demonstration of viral DNA integration into the genome of the cells, and [4] production of infectious viral stocks in cotton rat cells.

The amount of p24gag produced in the supernatants of cotton rat cells infected with HIV-1 stocks was in the order of the ng/ml and followed kinetics compatible with viral production. In addition we showed that the expression of the receptors on the cells determines the viral type specificity. Thus, CCR5 expressing cells were only infected by the R5-tropic strain BAL, whereas the CXCR4-expressing clone was only susceptible to the X4-tropic strain MN (Table 1).

We have readily detected viral episomal cDNA in HIV-1 infected cotton rat cells. However, it is the integration of the retroviral cDNA into the host chromosomal DNA that is the essential and distinctive step in viral replication. It has been shown that HIV-1 is able to integrate near repetitive sequences of the genome[22,23]. Although these are not the most favorable sites for integration[24], the insertion in their vicinity occurs most likely due to the abundance of these sequences in the genome (LINE 1 repetitive sequences are ~13% of the human genome). To demonstrate viral cDNA integration in cotton rat cells, we performed PCR reactions using a primer that annealed the LTR sequence of HIV-1 MN strain and a primer that annealed a repetitive LINE-1 like sequence in the cotton rat genome. With this set of primers we were able to isolate, clone, and sequence a fragment from infected cells that was a chimera between the LTRs sequence of HIV-1 and a LINE-1 repetitive sequence of the cotton rat [GenBank accession no. AY703985]. These data clearly demonstrate that the cotton rat cells, upon infection, are able to integrate HIV cDNA and use it as a template for replication.

Generation of HIV-1 viral stocks using clones of cotton rat cells expressing the human HIV-1 receptor complex was the final demonstration of productive replication in these cells. These stocks of virus infected human cells to similar extents as those stocks of virus produced in human cells, although with slight delay in the kinetics of p24gag production. This delay most likely represents a lower ratio of p24:infectivity and could suggest a less efficient assembly of progeny virions in cotton rat cells or the fact that the virus suffered some adaptations to the cotton rat system that rendered it less efficient in the subsequent infection of human cells.

There is currently a great effort focused on the development of microbicides to prevent sexually transmitted diseases (STDs). Epidemiological studies consistently demonstrated that recurrent infection by herpes simplex virus (HSV) increases the risk of HIV-1 acquisition and enhances HIV-1 replication[25,26]. PRO 2000, a synthetic naphthalene sulfonic polymer, interacts with viral glycoproteins gp120 of HIV and glycoprotein B of HSV-2 and has been tested as microbicide to prevent viral infection. PRO 2000 gel has been shown to inhibit vaginal simian/human immunodeficiency virus infection in macaques[27], HSV-2 infection in mice[28], gonorrhea in mice[29]. In recent clinical trials sponsored by NIAID (The HPTN 035 Study of Two Candidate Microbicides, BufferGel and PRO 2000 (0.5% dose)), PRO2000 was 30% more efficient than the placebo treatment to reduce HIV infection. We have previously developed a cotton rat model of vaginal HSV-2 infection[30]. Importantly, intravaginal pretreatment of PRO 2000 fully protected cotton rats against the challenge with HSV-2. These data indicate that microbicides active against STDs have been successfully tested in the cotton rat model.

## Conclusion

We have demonstrated that expression of HIV-1 receptors on the surface of cotton rat cells allows full cycle replication of the virus and further supports the potential of the cotton rat as a small animal model for the study of HIV-1 disease and pathogenesis. Efforts are currently being directed to the production of transgenic cotton rats *(Sigmodon sp*.) expressing human CD4, chemokine receptors, and human Cyclin T1 gene, on macrophages, microglia and T cells. Since this model has shown some low permissibility to HIV in the past, it will be important to determine how the expression of the human receptors in vivo will correlate with the data presented here.

## Methods

### Cells and culture conditions

The cotton rat cell lines CCRT (an osteogenic sarcoma) and VCRT (an undifferentiated spindle cell sarcoma) were isolated from inbred *S. hispidus *spontaneous tumors and they are routinely used in our laboratory[31]. Cells were cultured in Dulbecco Minimal Essential Media (D-MEM) with addition of 10% Fetal Calf Serum (FCS), 2 mM L-glutamine, 100 U/ml of penicillin, and 100 μg/ml of streptomycin. Cells were passed twice weekly and cultured at 37°C in a humidified atmosphere of 5% CO_2_.

### HIV-1 viral isolates

All virus isolates were from clade B of HIV-1[32] and were tissue culture adapted. The MN isolate was prepared and titrated in the human T lymphocyte cell line H9. The BAL and US1 isolates were prepared in the CCR5-transfected human acute lymphoblastic leukemia cell line A3/R5 and titrated in PHA-activated, CD8-depleted PBMC (human PBMC from an HIV-1 negative donor).

### Isolation of cotton rat cells expressing human CD4 and HIV-1 co-receptors

A clone of CCRT cells expressing hCD4 and hCXCR4 receptors (clone K4), was established by plasmid transfection. Briefly, the day before transfection parental CCRT cells were seeded into a 6-well plate at a density of 3 × 10^5 ^cell/well. The next day, the cells were transfected with a mixture containing the pCDNA3.1 expression vector for human CD4 (2 μg), and 6 μl of transfection reagent (FuGENE 6, Roche Molecular Biochemical). Stably transfected cells were selected by incubating the cultures in complete media with 800 μg/ml of geneticin (G418, Invitrogen). Small foci of resistant cells were subjected to positive selection using an anti-human CD4 antibody (monoclonal antibody RPA-T4, BD) and Dynabeads^® ^M450 conjugated with goat anti-mouse IgG (Dynal cat# 110.05). Subsequently, this pool of CCRT cells expressing hCD4 (4 × 10^6 ^cells) was electroporated with a mixture containing the pCDNA3.1 expression vector for hCXCR4 (10 μg), a selection plasmid (pGK-HygroB, 4 μg), and a carrier plasmid (pGEM-7zf +, 6 μg). Double transfected cells were selected in complete D-MEM media containing 800 μg/ml of G-418 and 500 μg/ml of hygromycin B and screened *in situ *for the expression of hCXCR4 with an anti-hCXCR4 monoclonal antibody (clone 12G5, IgG_2a_, κ, BD cat# 555974) and magnetic beads. Due to low levels of hCD4 expression in the CCRT K4 clone, most likely due to the silencing effect of chromatin on the integrated promoter, we enhanced the expression of the receptors by incubating the cell with the chromatin relaxing agent trichostatin A (TSA, 100 ng/ml)[33].

CCRT and VCRT cells stably expressing hCD4 and hCCR5 receptors were produced by transduction of both cell lines with infectious, replication-incompetent, retroviral particles encoding hCD4 and hCCR5 genes under the human cytomegalovirus immediate early promoter (pLCNX2 retroviral vector). Transduced cells were selected using G418 (800 μg/ml) and puromycin (300 μg/ml) and resistant clones were screened *in situ *for the expression of hCD4 and hCCR5 by antibody-conjugated to magnetic beads (hCD4, RPA-T4; anti-hCCR5, monoclonal antibody clone 2D7, IgG_2a_, κBD cat# 555993). Positive clones were further amplified and frozen.

### Infection of cotton rat cells

Filtered stocks (0.2 μM filter) of different HIV-1 isolates were used in all experiments. Cells were seeded in 24-well or 6-well plates at densities of 4 × 10^4 ^and 8 × 10^4 ^cells/well respectively, and allowed to attach for 24 h. In the case of the CCRT K4 clone, cells were induced with 100 ng/ml of TSA for additional 16 h[33] before infection. Stocks of the different isolates of HIV-1 (~1 and 2 × 10^4 ^TCID_50_) were added to each well and incubated for 4 h at 37°C. After infection, the cultures were washed several times with PBS, and cultured in fresh complete D-MEM overnight. Samples of supernatants were obtained at different times after infection and tested for the concentration of extracellular p24gag using a commercially available kit (p24 antigen assay kit from Coulter Immunology, cat# 6607051). For viral infectivity, PHA-activated, CD8-depleted PBMC or H9 cells were used as the recipient cells, and they were seeded at a density of 10^6 ^cells/well in a 24-well plate.

### Detection of HIV-1 cDNA and detection of integrated HIV-1 genome

CCRT K4 cells were seeded at a density of 8 × 10^4 ^cells per well of a six well plate, cultured for 24 h and induced with 100 ng/ml of TSA for an additional 16 h. The next day cells were infected with HIV-1 MN virus (1.6 × 10^4 ^TCID_50_) or with a mock viral preparation. Cells were incubated with the inocula for 4 h, washed with PBS, and incubated with fresh media for three additional days before using the monolayer for genomic DNA isolation using a Qiagen DNA isolation kit (Cat# 69504). Detection of HIV-1 cDNA was performed by PCR amplification using the following primers: (forward, 5' GGCTAACTAGGGAACCCACTGCTT 3'; reverse, 5' CCGAGTCCTGCGTCGAGAGAGC 3') and conditions (35 cycles with annealing temperature of 59°C). Equivalent dilutions of genomic DNA from CCRT K4 infected and mock-infected cell were used as input DNA in the PCR reactions. β-actin gene amplification was used as internal control and primers were previously described[34]. The expected products were confirmed by sequencing.

For detection of integrated HIV-1 genomes in cotton rat infected cells, a PCR reaction was designed using one primer targeting the Long Terminal Repeat (LTR) sequence of HIV-1 MN isolate (GenBank accession no. AF075719, 5' CTGTTCGGGCGCCACTGCTAGAG 3') and another targeting a repetitive DNA sequence in the cotton rat genome (LINE-1 retroposon ORF II pseudogene, GenBank accession no. AY041614, 5' AAAGAACAATACTCAATTTCATTTGG 3') using as reaction conditions 58°C for annealing and 2 minutes for extension time during 35 cycles.

## Competing interests

The authors declare that they have no competing interests.

## Authors' contributions

JB conceived the study and participated in its design, coordination, data analysis, and data preparation for publication. LP generated and characterized receptor-expressing cells. LW performed HIV-1 infections. DK generated some expression vectors for the receptors. TS characterized receptor expressing cells. CB provided with reagents, and with VP, and GP participated in the design of the study, analysis of the data, and preparation of the manuscript. All authors read and approved the final manuscript.
